# Experimental Investigation and Numerical Simulation for Corrosion Rate of Amorphous/Nano-Crystalline Coating Influenced by Temperatures

**DOI:** 10.3390/nano11123298

**Published:** 2021-12-05

**Authors:** Hamid Al-Abboodi, Huiqing Fan, Ibtihal A. Mahmood, Mohammed Al-Bahrani

**Affiliations:** 1 State Key Laboratory of Solidification Processing, School of Materials Science and Engineering, Northwestern Polytechnical University, Xi’an 710072, China; hamid80_n88@yahoo.com; 2 Department of Mechanical Engineering, University of Technology, Baghdad 190006, Iraq; ibtihalnamie@yahoo.com; 3 School of Engineering, University of Plymouth, Plymouth PL4 8AA, UK; mohammed.naeem@plymouth.ac.uk; 4 Research and Studies Unit, Al-Mustaqbal University College, Hillah 51001, Iraq

**Keywords:** HVOF, corrosion rate, vacuum heat treatment, pitting corrosion, COMSOL

## Abstract

A high-velocity oxygen fuel (HVOF) system was employed to prepare a Fe_49.7_Cr_18_Mn_1.9_Mo_7.4_W_1.6_B_15.2_C_3.8_Si_2.4_ amorphous coating on mild steel. The electrochemical behavior of the resultant coatings, namely as-sprayed coating and vacuum heat-treated coating (at 650 °C and 800 °C), were investigated in a 3.5% NaCl solution at variable temperatures using scanning electron microscopy (SEM), electrochemical impedance spectroscopy (EIS), potentiodynamic polarization, optical microscopy (OM), and XRD diffraction. Moreover, COMSOL Multiphysics version 5.5 software were employed for predicting the galvanic corrosion of amorphous material immersed in an aqueous NaCl solution, using the software finite element kit. The experiments demonstrated that the coatings’ pitting resistance was significantly affected by temperature. The results also showed that temperature affected the pitting corrosion rate and changed the shape of the pits. However, the changes were not as extreme as those observed in stainless steel. Furthermore, there was no significant difference between the as-sprayed coating and the vacuum-heat-treated coating at 650 °C. At low NaCl concentrations at and temperatures below the critical pitting temperature, the resulting pits were significantly small with a hemisphere-like. By contrast, at a higher NaCl concentration at 70 °C, particularly in the case of heating at 650 °C, the pits appearing on the Fe-based amorphous coating were vast and sometimes featured a lacy cover.

## 1. Introduction

Fe-based bulk metallic glasses (BMGs) have received significant interest in recent decades because of their superior performance, such as their high strength and hardness, good magnetic properties, and excellent corrosion and wear-resistance [1,2,3,4,5,6,7,8,9]. Compared to stainless steel and crystalline alloys with a similar composition, BMG boasts a uniform microstructure without any crystal imperfections, dislocations, or grain limits. Hence, they are leading coating products for marine and industrial applications [10]. However, pitting corrosion triggered by airborne-sea-salt deposits in marine environments is a matter of great concern [11,12]. Utilizing amorphous coatings as surface protection for marine components is an effective solution to alleviate such problems. These coatings are also extremely useful for protecting steel structures (e.g., communication towers, bridges, floodgates, underground cable supports, and underground radioactive waste disposal sites) from continued exposure to severe open-air conditions [13]. As mentioned previously, Fe-based amorphous alloy products are among the most effective amorphous alloys considering their unique properties. In thermal spray coating [14,15,16,17], the amorphous structure is conserved because of the coating’s fast cooling, which inhibits long-range diffusion and crystallization. The method can be used for steel substrate coating applications. This method enhances the properties of steel surfaces, such as improved corrosion resistance compared to chrome plating and stainless-steel in NaCl solution [18,19]. Several studies have investigated the performance of amorphous coatings subjected to corrosion agents [20,21,22,23,24,25,26,27,28]. Bakare et al. [20] investigated crystalline and amorphous forms of the FeCrMoCB alloy in 0.5 M H_2_SO_4_ and 3.5% NaCl solution. They concluded that the enhanced corrosion resistance of the amorphous alloys could have been due to their homogeneity, mainly through the exclusion of the Mo-rich phase. Naka et al. [22] conducted electrochemical studies on amorphous Fe-Cr-P-C and Fe-Cr-Ni-P-C alloys immersed in acid and neutral-chloride solutions. The results showed that pitting corrosion did not appear on the surface of the amorphous Fe-based alloys. The authors concluded that the superior corrosion resistance of the amorphous Fe-based alloys is associated with chromium and phosphorus and the homogeneous amorphous structure of the single-phase. Zhang et al. [26] scrutinized the FeCrMoCBY coating corrosion resistance in an acidic NaCl electrolyte. The electrochemical tests revealed that the coatings subjected to the NaCl solution featured a comparatively low passive-current-density and a significantly sizeable passive region, exhibiting higher resistance to localized corrosion. In this paper, the effect of different temperatures on the corrosion rate of the Fe_49.7_Cr_1.8_Mn_1.9_Mo_7.4_W_1.6_B_15.2_C_3.8_Si_2.4_ amorphous coating was studied. An amorphous coating prepared using the HVOF method was subjected to annealing heat treatment to examine the various crystallization states. Electrochemical tests in the simulated marine environment (3.5% NaCl solution) were used to measure and compare the different coatings’ corrosion rates. Furthermore, COMSOL Multiphysics software was employed to numerically simulate the experimental results.

## 2. Experimental Procedure

### 2.1. Material and Coating Preparation

**Figure 1:**
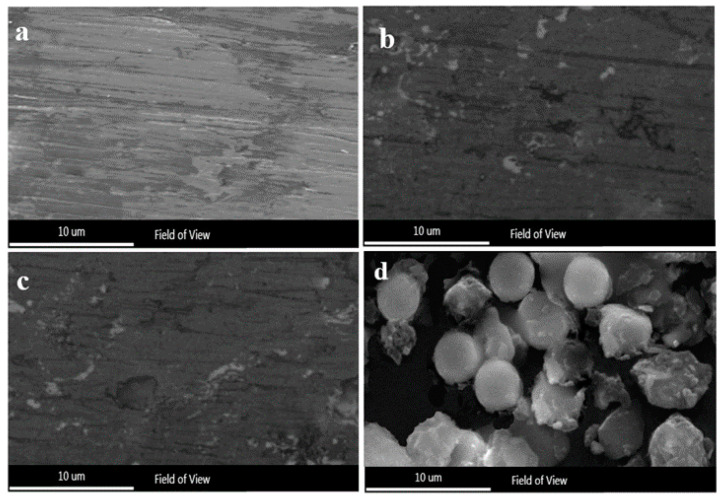
Optical micrograph of the (**a**) as-spray coating, (**b**) coating with heat 650 °C and (**c**) coating with heat 800 °C; (**d**) SEM image of Fe_49.7_ Cr_18_ Mn_1.9_ Mo_7.4_ W_1.6_ B_15.2_ C_3.8_ Si_2.4_ alloy powders.

Mild steel with dimensions of 100 mm × 150 mm × 5 mm was chosen as a substrate. A masterbatch of Fe_49.7_Cr_1.8_Mn_1.9_Mo_7.4_W_1.6_B_15.2_C_3.8_Si_2.4_ percent and 99.9% were prepared from Fe, Cr, Mn, Mo, W, B, C, and Si mixtures. The thermal spray constituted an amorphous powder with a particle size of 25–45 μm. Optical microscopy (OM OM; CX23, Olympus, Tokyo, Japan) presents the microstructure of the coatings, as shown in Figure 1a–c. An HVOF spraying device (Met-Jet 4l, Metallisation, West Midlands, UK) was prepared to create a powder coating with a thickness of 300 μm. The spraying parameters optimized and used in another experiment are provided in Table 1. Utilizing wire electrical discharge machining (Partline Limited, West Yorkshire, UK), several square-shaped samples were removed following the spraying process to prepare the specimens required for microscopic inspection and electrochemical corrosion testing. Using diamond abrasive paper, the resulting specimens were polished to a surface roughness of Ra = 0.1 μm for electrochemical corrosion testing. Furthermore, the specimens were ground to a cutting-edge of >1 mm for microscopic examination. The latter processing of the specimens was performed to circumvent the heat-affected area on the coating microstructure reduced by the WEDM. Finally, the specimens were subjected to vacuum heat treatment at 650 °C and 800 °C, both for 4 h, to correspondingly examine the thermodynamic stability and crystallization temperature of the Fe-based amorphous coatings.

### 2.2. Microstructure Characterization

The powdered microstructure and sprayed coatings (without heat treatment, vacuum heat treated at 650 °C, and vacuum heat treated at 800 °C) were examined using scanning electron microscopy (SEM; 6700F, JEOL, Tokyo, Japan). The specimens were inspected using an X-ray diffractometer device (XRD; X’ Pert PRO MPD, Philips, Eindhoven, Netherlands) with Copper K-α radiation before and after heat treatment. X-ray diffraction was initiated with a 2 h diffraction angle varying from 20 to 80 degrees. The resulting optical microscopy (OM) images were analyzed to assess the porosity proportion in the coatings. An oxygen/nitrogen analyzer was employed to determine the oxygen content of the coatings and powders. 

### 2.3. Corrosion Tests

The electrochemical tests were conducted using a PARSTAT4000A electrochemical workstation (PAR, PARSTAT4000A, Advanced Measurement Technology, Inc., Oak Ridge, TN, USA) equipped with Versa Studio v2.4 software from Princeton Applied Research^®^, Advanced Measurement Technology Inc., Oak Ridge, TN, USA. Samples with a 1 cm^2^ exposure area were selected as working electrodes. Furthermore, platinum sheets were selected as the counter electrodes, and a saturated calomel electrode (SCE) was used as the reference electrode. A 3.5% NaCl-based solution was employed as a corrosive medium for the electrochemical tests. A combination of NaOH and HCl adjusted the NaCl solution pH. Finally, three pH = 1, pH = 7, and pH = 13 solutions were prepared to simulate the strong acid (pH = 1), neutral (pH = 7), and strong alkali (pH = 13) corrosive behavior conditions, respectively. The electrochemical tests were conducted at 30 °C, 40 °C, 50 °C, 60 °C, and 70 °C. Potentiodynamic polarization curves were obtained at a 0.5 mV/s scan rate. It is noteworthy that the specimens were fully immersed for 30 min before testing. EIS with a signal amplitude of 10 mV and frequency range of 1 × 10^5^ to 1 × 10^−2^ Hz was used to check the corrosion rate of specimens throughout the 30 min long immersion. Cview software version 12.23 (Chelsea Technologies Ltd., Molesey, UK) was used to fit and evaluate the polarization curves. Meanwhile, ZSimpWin software version 3.2 (AMETEK, Inc., Oak Ridge, TN, USA) was used to fit and evaluate the EIS curves. More than three samples were tested each time to ensure the reproducibility of the results.

## 3. Results and Discussion

### 3.1. Microstructural Characterization

**Figure 2:**
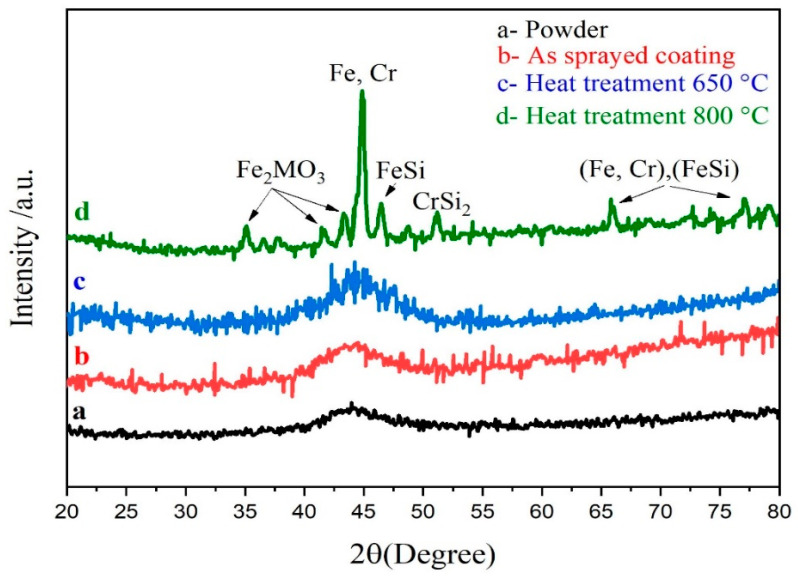
X-ray diffractometer (XRD) spectrum of (**a**) powder, (**b**) as-sprayed coating, (**c**) coating specimens by heat treatment at 650 °C for 4 h and (**d**) 800 °C for 4 h.

The SEM images of the Fe_49.7_Cr_18_Mn_1.9_Mo_7.4_W_1.6_B_15.2_C_3.8_Si_2.4_ feedstock powders with 25–45 μm diameters are shown in Figure 1d. Most of the particles formed in the argon atmosphere using gas atomization are spherical or near-spherical. Some particles feature tiny satellites attached to them, but most feature smooth surfaces that illustrate good fluidity. Figure 2 depicts the XRD diffraction of an Fe-based amorphous powder and coating sprayed using the HVOF system. No significant Bragg diffraction peak (crystal peak) was observed. However, according to Figure 2a, a low diffusion peak was observed at 2θ = 44°, signifying that the Fe_49.7_Cr_18_Mn_1.9_Mo_7.4_W_1.6_B_15.2_C_3.8_Si_2.4_ powder’s degree of amorphization was very high. Compared to the amorphous powder, using the HVOF system, the Fe-based amorphous coating diffusion peak intensity improved considerably. However, according to Figure 2b, no crystallization peak appeared. The microstructure of the coating deposited using the HVOF device was both amorphous and nanocrystalline. Therefore, according to previous research [27], it can be concluded that the diffusion peak at 2θ = 44° was a diffraction from the nanocrystalline phase within the coating. Furthermore, following heat treatment at 650 °C, the XRD spectrum (Figure 2c) of the coating retained a diffuse peak, denoting that nanocrystalline growth was insignificant. However, considering that the Bragg diffraction peaks in the XRD diffraction pattern were distinct, the amorphous coating crystallized at 800 °C (Figure 2d). The experiment revealed that the HVOF deposition process is an effective technique for preparing amorphous coatings, practically for maintaining the powder initial amorphous structure. Furthermore, it was shown that the amorphous Fe-based coating can work under 650 °C without any phase structure changes.

### 3.2. Corrosion Rate in NaCl Solution 

**Figure 3:**
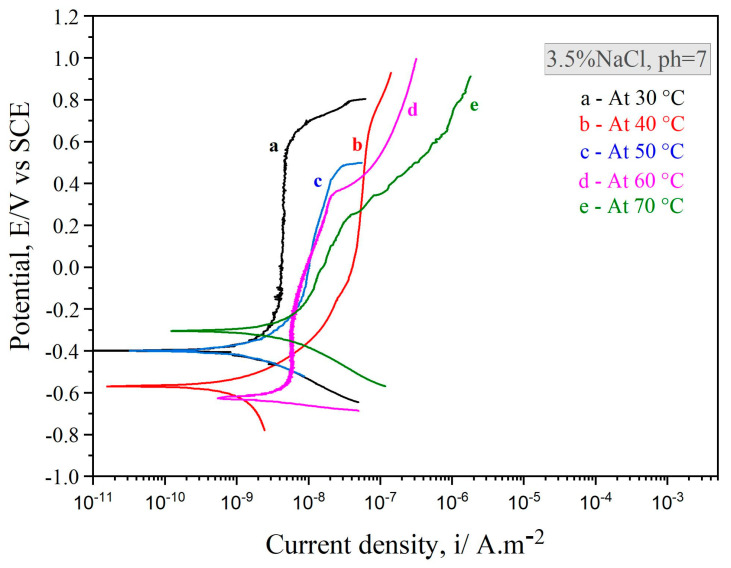
Potentiodynamic polarization curves of the Fe_49.7_Cr_1.8_Mn_1.9_ Mo_7.4_W_1.6_B_15.2_C_3.8_Si_2.4_ coatings deposited under the conditions of 30 °C, 40 °C, 50 °C, 60 °C, and 70 °C in 3.5% NaCl solution.

The Fe_49.7_Cr_18_Mn_1.9_Mo_7.4_W_1.6_B_15.2_C_3.8_Si_2.4_ coating potentiodynamic polarization curves at variable temperatures in a 3.5% NaCl solution are shown in Figure 3. Despite the gradual atomization of the passive film, the amorphous coatings at 30 °C, 40 °C, and 50 °C demonstrated comparable polarization behavior and were passivated with low passive-current-density. They also featured a sizeable passive zone, suggesting that the coatings were resistant to localized corrosion. Furthermore, the polarization current density did not change dramatically as a function of the passivation film formed on the samples until the potential reached the pitting potential. At this point, the current density rapidly increased, meaning that pitting corrosion or passivation film breakdown could occur. When the potential was lower than the pitting protection potential, the current density was lower than the passivation current density, suggesting that the passivation film had been returned to its passivation state and that pitting had been eliminated. In the marine environment, pitting corrosion is the most frequent failure mode of passivated metal materials. The creation of pits would dramatically decrease the metal component service life. Compared to the exact coating at varying temperatures, this indicated a robust passive film on the as-sprayed coating surface and higher corrosion resistance at temperatures 30 °C, 40 °C, and 50 °C. Therefore, it can be posited that the microstructure created by the spraying parameter has a significant impact on corrosion resistance in amorphous coatings. Furthermore, different pH values of corrosive media influenced the Fe-based amorphous coating electrochemical polarization’s behavior. A passive film was formed on the Fe-based amorphous coating after being polarized in an extremely acidic (pH = 1) NaCl solution. Nonetheless, the results showed that the passive film chemical stability was inferior to the film formed in the neutral solution. Besides, complete or durable passive films are difficult to form under extremely alkaline environments, which reduce amorphous/nano-crystalline coatings’ corrosion resistance [29].

**Figure 4:**
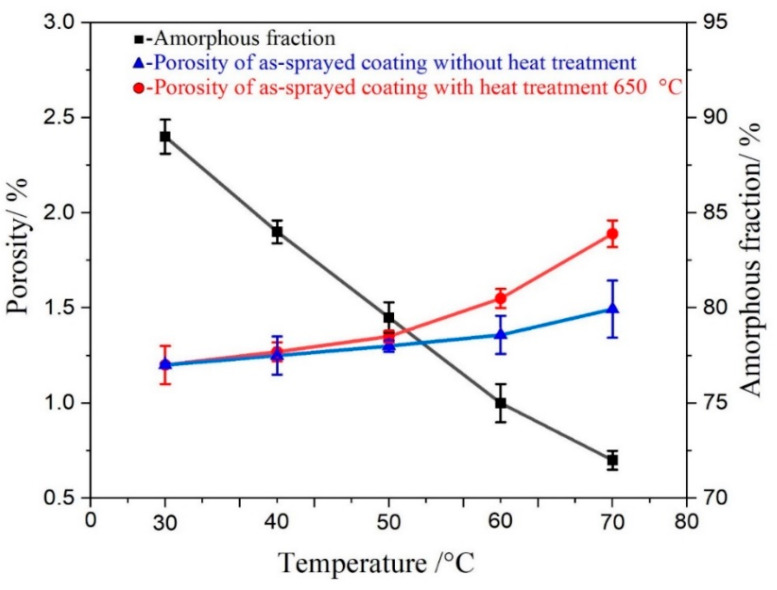
Comparison of the porosity and amorphous phase fraction of the coatings deposited under the conditions of 30 °C, 40 °C, 50 °C, 60 °C and 70 °C.

Amorphous coatings’ corrosion resistance increase with lower porosity or higher amorphous phase material with the same composition [30]. Nonetheless, in this analysis, the two variables followed the same pattern as seen in Figure 4. According to the corrosion test findings, we assumed that the two influences carried the primary position across various ranges. Because of pore removal, the coatings’ corrosion resistance meaningfully increased as the porosity declined by 1.22% for the as-sprayed coating at 30 °C, and by 1.89% for the as-sprayed coating at 70 °C. Low porosity and amorphous phase correspond to high corrosion resistance. However, the resistance declined with higher temperatures because of the breakdown of the surface passive film layer. Because of the more uniform passive surface layer shape and low porosity, the corrosion resistance tended to be more resilient to amorphous phase material, leading to the as-sprayed coating performing better than the other coatings at 30 °C. 

**Figure 5:**
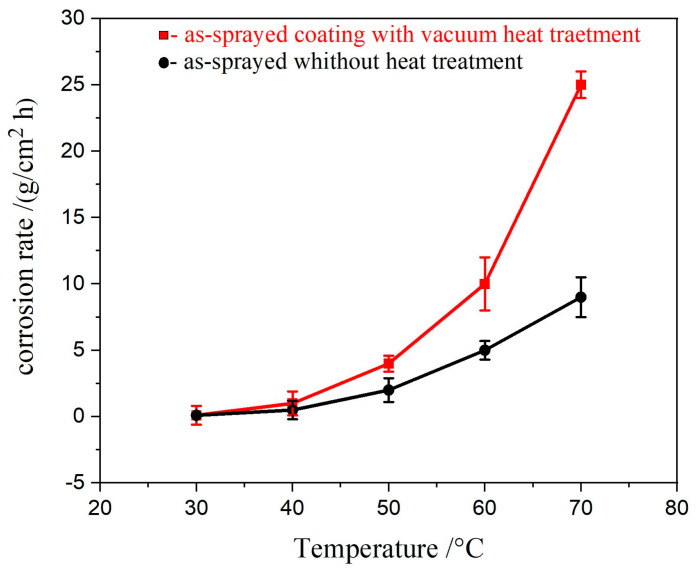
Relationship between temperature and corrosion rate of as-sprayed coating with vacuum heat treatment at 650 °C and as-sprayed coating without heat treatment.

Consequently, the optimal spraying parameter for all thee specimens was to prepare Fe_49.7_Cr_18_Mn_1.9_Mo_7.4_W_1.6_B_15.2_C_3.8_Si_2.4_ amorphous coating with proper porosity and amorphous phase content, thereby achieving the best corrosion resistance. However, at high-temperature conditions, the coatings’ ability to resist localized corrosion (e.g., pitting) steadily deteriorated. Figure 5 illustrates a Fe_49.7_Cr_18_Mn_1.9_Mo_7.4_W_1.6_B_15.2_C_3.8_Si_2.4_ coating that was split into two cases. The corrosion rate increased smoothly in the first case (coating without heat treatment) but rose rapidly in the second phase (coating with heat treatment at 650 °C). As a result, it can be said that the corrosion rate accelerated when the temperature reached 45 °C. 

**Figure 6:**
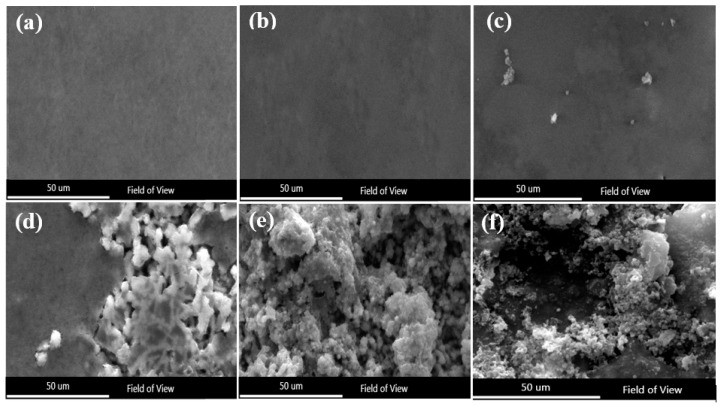
SEM images of pitting formed on the samples after test: (**a**) as-sprayed coating, (**b**) 3.5% NaCl, 30 °C, (**c**) 3.5% NaCl, 40 °C, (**d**) 3.5% NaCl, 50 °C, (**e**) 3.5% NaCl, 60 °C, (**f**) 3.5% NaCl, 70 °C.

The morphologies of the amorphous coatings surfaces at varying temperatures after electrochemical analysis are shown in Figure 6. As shown in Figure 6b, the amorphous coating did not suffer from pitting corrosion and featured morphologies close to the pre-testing morphologies, shown in Figure 6a. Although pitting occurred as the temperature increased, it exhibited general corrosion activity before the chromium was transpassively dissolved [9]. The number of pits increased dramatically when the solution temperature exceeded 50 °C. Furthermore, the density followed the same pattern. In addition, the pit shapes (the diameter of the pit increases) forming at 30 °C and 40 °C were different. As depicted in Figure 6e, when the temperature reached 60 °C, the pits became a deep depression in the ground and formed a massive hole with a visible lacy cover. As seen in Figure 6f, when the temperature reached 70 °C, the cover became finely perforated and consistent with a rugged interior and dish-shaped profiles. A pit is formed according to the following steps: first, early pit growth is hemispheric, with pit contents covered by perforated residue from the passive film. The pit cover is then broken when it approaches a critical height, leaving an exposed hemispheric cavity. Even if passivation is not a problem, the open hemisphere-shaped pit cavity is a temporary form as the rising pits transform into saucer-shaped cavities under anodic diffusion regulation.

**Figure 7:**
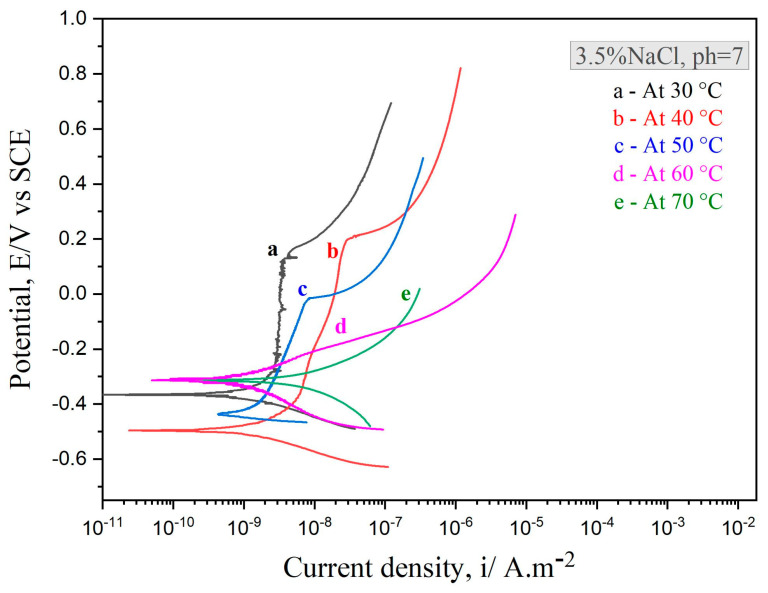
Potentiodynamic polarization curves of the Fe_49.7_Cr_1.8_Mn_1.9_ Mo_7.4_W_1.6_B_15.2_C_3.8_Si_2.4_ coatings with vacuum heat treatment at 650 °C under the conditions of 30 °C, 40 °C, 50 °C, 60 °C, and 70 °C in 3.5% NaCl solution.

**Figure 8:**
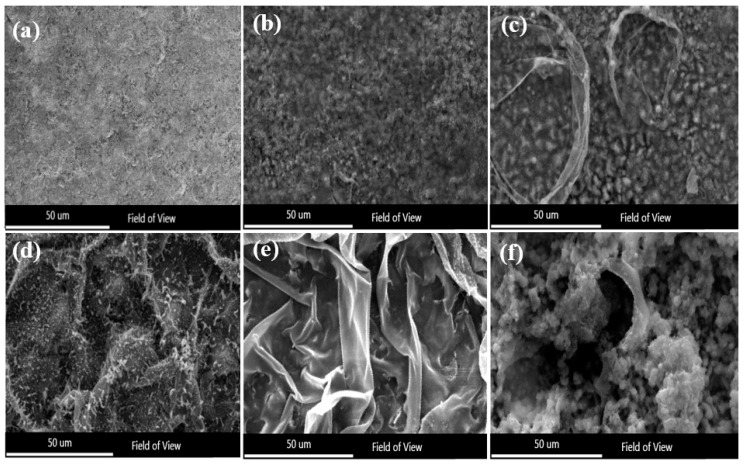
SEM images of pitting formed on the samples (heated 650 °C) after test: (**a**) as-sprayed coating, (**b**) 3.5% NaCl, 30 °C, (**c**) 3.5% NaCl, 40 °C, (**d**) 3.5% NaCl, 50 °C, (**e**) 3.5% NaCl, 60 °C, (**f**) 3.5% NaCl, 70 °C.

The electrochemical polarization test for as-sprayed coatings with vacuum heat treatment at 650 °C was investigated at various temperatures, as shown in Figure 7. The density of the passive film was calculated to be equal to that of an as-sprayed coating without vacuum heat treatment, but the destruction of the passive film and the rate of corrosion were faster. In addition, the passive film appearing on the heat-treated coating was more turbulent than that of the as-sprayed coating. The reason for this disturbance is the effect of pre-treatment, which affects the behavior of the coating. Interestingly, pre-heating the coating affected the destruction of the passive film. It can be seen from Figure 7 that the disturbance increased when the temperature increased faster than in the case of the coating without heat treatment. Following the electrochemical analysis, the surface morphology of the amorphous coatings (vacuum heat-treated at 650 °C) with varying temperatures can be seen in Figure 8. It was shown that the amorphous coatings subjected to vacuum heat treatment at 650 °C did not suffer from pitting corrosion (i.e., their secondary morphology was close to their initial morphology before treatment). This was not the case for the untreated coatings, as certain peels occurred on the surface. The presence of a peeling condition on the surface of the coatings suggests that the passive film gradually weakens and decomposes. The peels started when the coating was exposed to 40 °C in NaCl solution, as shown in Figure 8c. Whenever the temperature increased, the peels increased until they changed to a hole-like dish inside the coating surface, as shown in Figure 8f.

### 3.3. EIS Measurements

**Figure 9:**
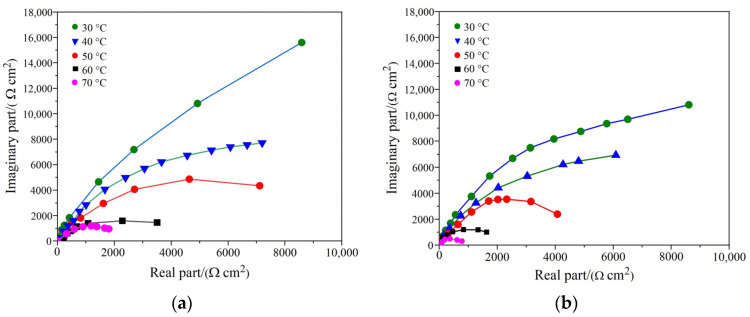
(**a**) Nyquist plots of as-sprayed coating in 3.5% NaCl solution; (**b**) Nyquist plots of coating (without vacuum heat treatment 650 °C) in 3.5% NaCl solution.

Electrochemical impedance spectroscopy was used to assess the differences between the amorphous coatings’ electrochemical reactions. Figure 9a,b depict the Nyquist plots for the as-sprayed and heat-treated coatings in 3.5% NaCl solution at the open-circuit potential. Coating at 30 °C indicates a single time constant, and a straight line with an approximate 45 degree slope occurs at low frequency, implying the formation of a Warburg impedance. A semi-infinite duration diffusion mechanism impacts the charge transfer [31]. It was established that diffusion could be associated with oxide breakdown at interparticle boundaries in the coating, creating diffusion channels, leading to inner corrosion [31]. On the other hand, the Nyquist plots at 60 °C and 70 °C show two continuous semi-circles representing two-time constants. The behavior indicates that the increase in temperature influences coating surfaces.

**Figure 10:**
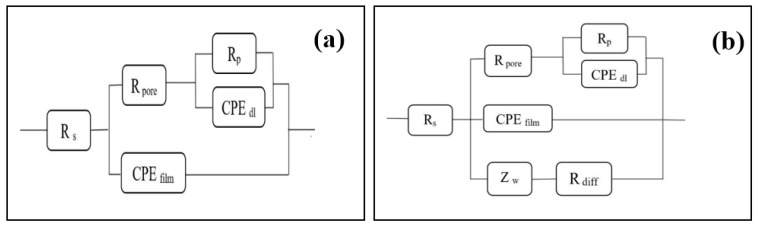
Equivalent circuits proposed for fitting of EIS plots: (**a**) model for single corrosion mechanism of pitting corrosion; (**b**) model for double corrosion mechanism of pitting corrosion.

Based on the aforementioned analysis, Figure 10a,b show the corresponding circuits that explain the electrochemical reaction for the amorphous coatings. Here, Rs denotes electrolytic resistance; CPE**_film_** signifies the capacitance properties of the passive film formed on the coating; R**_pore_** is in-hole resistance; Rp is polarization resistance; and CP**_Edl_** represents interfacial capacitance at the corrosion points on the passive film. By contrast, according to Figure 10b, the crystallized coating circuit has two additional components. Warburg impedance (Z**_w_**) and resistance (R**_diff_**) are considered for simulating the active dissolution zone impedance of the coating. The associated parameters of the equivalent circuits and the open circuit potential (OCP) are summarized in Table 2.

## 4. Model of Corrosion Rate

**Figure 11:**
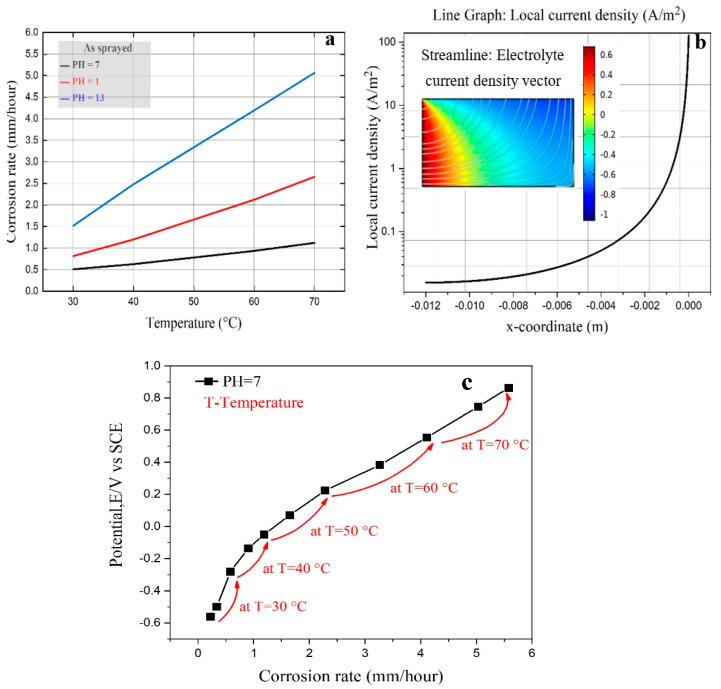
(**a**) Corrosion rate with various temperatures, (**b**) local current density vs. disc radii for 3.5% NaCl electrolyte and (**c**) the relationship between corrosion potential and corrosion rate.

The model was developed in COMSOL Multiphysics version 5.5 software with a specific electrolyte domain to investigate the corrosion rate of the amorphous coating under various temperatures in 3.5% NaCl solution, as shown in Figure 11a. The model can be used with the experimental results, as indicated in Figure 11c, to demonstrate the amorphous coatings’ galvanic corrosion prediction. An increase in temperature and concentration increases the critical current density while having little effect on Ip and Epp. The addition of chloride ions to the Fe_49.7_Cr_18_Mn_1.9_Mo_7.4_W_1.6_B_15.2_C_3.8_Si_2.4_ amorphous coating has a similar effect. It can be observed that higher temperatures can aggravate metal corrosion. Corrosion can be exacerbated by elevated acidity and peroxide value following oxidation. Figure 11b reveals the influence of the disc radii on the local current density for coating. It can be observed that as the disc radius increased, the local current density increased. This may be attributed to corrosion dimensional expansion in the amorphous coating electrode. This influence, on the other hand, would be visible from the edges, so the local current density dropped drastically when approaching the disc boundaries. The findings revealed that flaws or edges are possible corrosion initiation points. Furthermore, the streamline of the electrolyte current density for the coating surface can be observed. Since the Nernst equation states that the equilibrium potential for oxygen reduction is dependent on pH, the general equation for pH = 7 in this model is:

(1) $${\;E}_{\mathrm{eq},o2}=1.23V-\frac{\mathrm{RT}}{4F}\mathrm{Ln}\left(\frac{1}{0.21\times 10^{-\mathrm{pH}}}\right)$$

where (T) is the temperature, (R) is the universal gas constant, and (F) is the Faraday constant. Furthermore, the equation of heat transfer for coating in this model is:

(2) $$D_{z}{\rho C}_{p}u\nabla T+\nabla q=D_{z}Q\;$$

where (∇T) is the overall temperature difference, and (Q) is the rate heat of transfer. The q, the local heat flux density, is:

(3) $$q=-D_{z}k\nabla T\;$$

where (q) is the local heat flux density. 

To calculate the corrosion rate, we can use the following equation:

(4) $$\nabla j+u\nabla C_{i}=R\;$$

where (R) is the corrosion rate, (j) is the flux, (u) is the velocity, and (C_i_) is the concentration.

## 5. Conclusions

Amorphous coatings passivate spontaneously in chloride solutions. The corrosion rate in 3.5% NaCl increased along with the increase in the temperature of the solution. It was noted that the corrosion occurred at a faster rate when the temperature was high. As the concentration of NaCl or the temperature of the solution increased, the pitting potential of the Fe_49.7_Cr_18_Mn_1.9_Mo_7.4_W_1.6_B_15.2_C_3.8_Si_2.4_ coating moved towards the more active direction, representing a drop in corrosion resistance and a higher possibility of pitting corrosion. Corrosion current density generally increases, showing a linear relationship with temperature rise. Passive film thickness and pit size are temperature-dependent. The results demonstrated that when the NaCl temperature was low, the resulting pits were thin, sparse, and hemisphere-like. Higher temperatures, on the other hand, caused pits to emerge on the lacy cover. The lacy cover on the surface degraded in response to the rise in temperature, leaving a vast void with dish-shaped profiles and a raw interior surface. According to the analyzed results, the Fe_49.7_Cr_18_Mn_1.9_Mo_7.4_W_1.6_B_15.2_C_3.8_Si_2.4_ amorphous material can be used in a marine environment at temperatures below 60 °C, but coatings without vacuum heat treatment demonstrate a better performance compared with vacuum-treated coatings. The results of the numerical simulation of the COMSOL software were in close agreement with the experimental results, in terms of the effect of the temperature on the coatings’ corrosion behavior.

## Figures and Tables

**Table 1:** Spraying parameters employed in the HVOF process.

Parameter	Condition
Spraying distance, mm	380
Feed rate, g/min	100
Transverse speed, mm/s	10,000
Oxygen, NLPM	835
Kerosene flow, mL per min	260
HVOF gun nozzle, cm	10
Carrier gas (argon), L/min	9
Combustion pressure, Bar	8.5

**Table 2:** The fitting parameters of EIS in neutral NaCl by the proposed equivalent circuit (EC) in Figure 9a,b.

Samples	R_s_ (cm^2^)	R _pore_ (cm^2^)	R_p_ (cm^2^)	CPE film		CPE dL	
				Y_0_ (cm^−2^ s^−n^)	n	Y_0_ (cm^−2^ s^−n^)	n
As-sprayed coating	14.91	1561	1.7 × 10^5^	2.77 × 10^−5^	0.89	3.39 × 10^−4^	0.44
Coating with heat treatment 650 °C	14.96	1060	3.7 × 10^11^	1.84 × 10^−5^	0.91	2.24 × 10^−4^	0.35

## Data Availability

The code is available under the National Nature Science Foundation (51672220), the SKLSP Project (2019-TZ-04) of China, and the Fundamental Research Funds for the Central Universities of NPU (3102019GHXM002).
